# Supplementary material for: Low Serum Levels of Soluble Receptor Activator of Nuclear Factor κ B Ligand (sRANKL) Are Associated with Metabolic Dysregulation and Predict Long-Term Mortality in Critically Ill Patients
Source: Diagnostics (Basel). 2021 Dec 28;12(1):62. doi: 10.3390/diagnostics12010062 (PMC8774641; doi:10.3390/diagnostics12010062)
Supplement: Supplementary file 1 [file diagnostics-12-00062-s001.zip › diagnostics-1475771-SI.pdf]

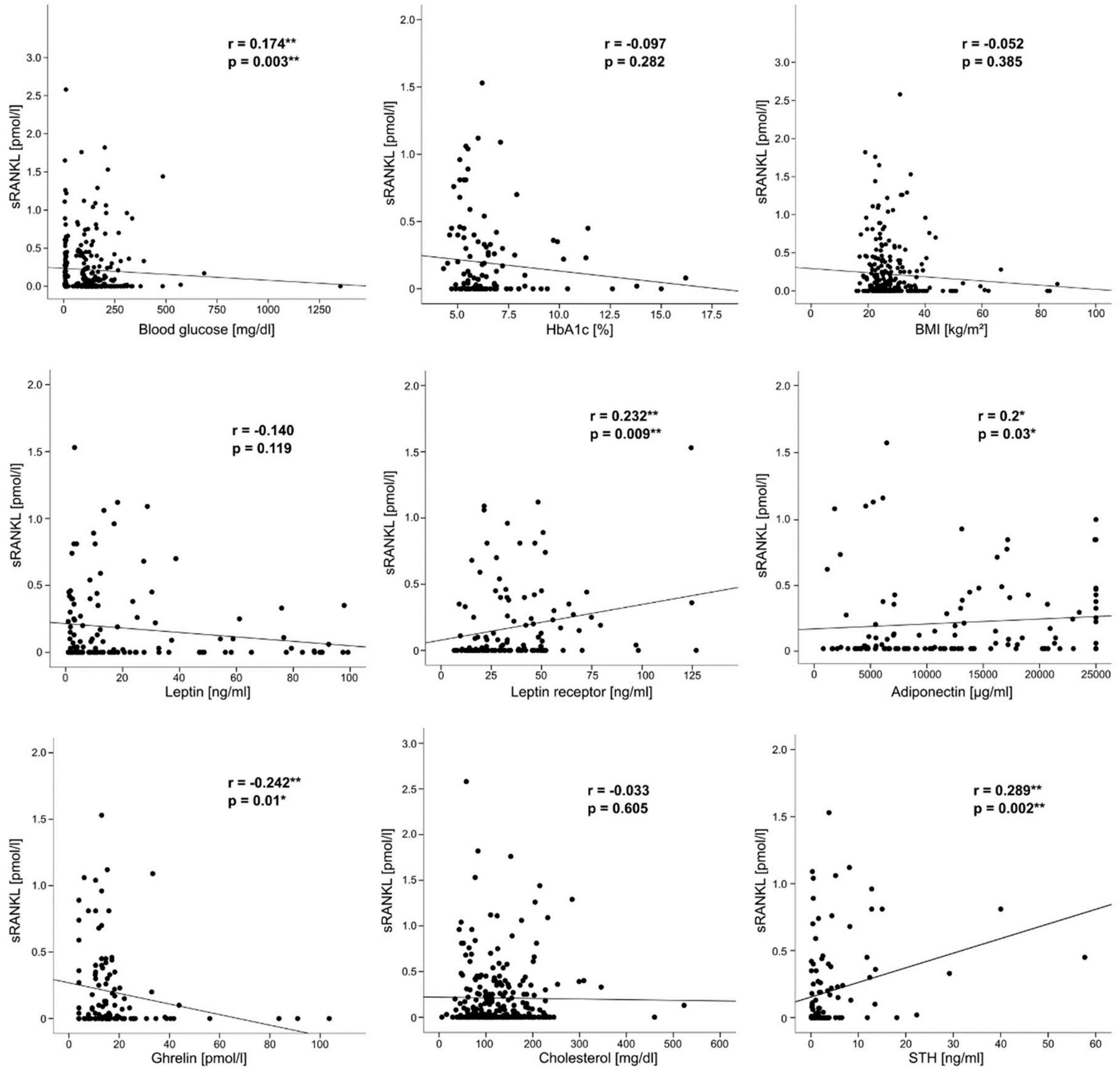

Figure S1. sRANKL serum levels are associated with features of diabetes and metabolic dysfunction. Correlation analyses of sRANKL serum levels with features of metabolic dysregulation (Blood glucose, HbA1c, BMI, Cholesterol) as well as adipokines (Leptin, Leptin receptor, Adiponectin) and growth factors (Ghrelin, STH). All data are shown as available. * $p < 0.05$; ** $p < 0.005$.
